# Niche Differentiation of Host-Associated Pelagic Microbes and Their Potential Contribution to Biogeochemical Cycling in Artificially Warmed Lakes

**DOI:** 10.3389/fmicb.2020.00582

**Published:** 2020-04-23

**Authors:** Md Sainur Samad, Hyo Jung Lee, Slawek Cerbin, Marion Meima-Franke, Paul L. E. Bodelier

**Affiliations:** ^1^Department of Microbial Ecology, Netherlands Institute of Ecology, Wageningen, Netherlands; ^2^Department of Biology, Kunsan National University, Gunsan, South Korea; ^3^Department of Hydrobiology, Faculty of Biology, Adam Mickiewicz University, Poznań, Poland

**Keywords:** microbiome, niche differentiation, zooplankton, bacterioplankton, phytoplankton

## Abstract

It has been proposed that zooplankton-associated microbes provide numerous beneficial services to their “host”. However, there is still a lack of understanding concerning the effect of temperature on the zooplankton microbiome. Furthermore, it is unclear to what extent the zooplankton microbiome differs from free-living and particle-associated (PA) microbes. Here, we explicitly addressed these issues by investigating (1) the differences in free-living, PA, and zooplankton associated microbes and (2) the impact of temperature on these microbes in the water column of a series of lakes artificially warmed by two power plants. High-throughput amplicon sequencing of the 16S rRNA gene showed that diversity and composition of the bacterial community associated to zooplankton, PA, and bacterioplankton varied significantly from one another, grouping in different clusters indicating niche differentiation of pelagic microbes. From the abiotic parameters measured, temperature significantly affected the diversity and composition of all analyzed microbiomes. Two phyla (e.g., Proteobacteria and Bacteroidetes) dominated in zooplankton microbiomes whereas Actinobacteria was the dominant phylum in the bacterioplankton. The microbial species richness and diversity was lower in zooplankton compared to bacterioplankton and PA. Surprisingly, genera of methane-oxidizing bacteria, methylotrophs and nitrifiers (e.g., *Nitrobacter*) significantly associated with the microbiome of zooplankton and PA. Our study clearly demonstrates niche differentiation of pelagic microbes and their potential link to biogeochemical cycling in freshwater systems.

## Introduction

In the pelagic environment, zooplankton is widely distributed and represents an important component of aquatic food webs. They consume phytoplankton and keep the water column clear (suppress blooming) (Pogozhev and Gerasimova, 2001; Gerasimova et al., 2018) and are an important link to higher trophic levels of the food web. For example, zooplankton is one of the main food sources for fish. Like plants and higher animals, zooplankton harbor a rich bacterial community, as zooplankton bodies are nutrient-rich micro-environments that provide nutrients for rapid growth of bacteria (Newton et al., 2011). Bacterial colonization of zooplankton has been proposed to provide numerous benefits including nutrient acquisition, stress protection, detoxification, and habitat provision (Tang et al., 2010). Colonization by bacterial species can be observed throughout the body of zooplankton, especially around the oral region, the anus, body appendages, intersegment parts, and the intestine (Huq et al., 1983; Carman and Dobbs, 1997). Hence, the whole body of zooplankton constitutes “hotspots” for many known and unknown microorganism that provide numerous ecosystem services. However, warming can be a potential threat for zooplankton’s fitness (Kessler and Lampert, 2004; Pajk et al., 2012). In addition, it is still not known how this warming affects the host-associated microbiome (e.g., zooplankton and phytoplankton) in relation to the free-living bacterioplankton. To explore this, we investigated a set of natural, artificially warmed-lakes.

Copepods (e.g., Cyclopoid and Calanoid), cladocerans (e.g., *Bosmina* and *Daphnia*), and rotifers are composed of the meso- and microscopic groups of zooplankton. They are highly abundant in the pelagic environment of nearly all freshwater as well as marine habitats where they consume a wide range of food (e.g., phytoplankton and bacterioplankton) for their growth. Parameters that influence their growth include various abiotic factors, such as temperature (Huntley and Lopez, 1992; Ikeda et al., 2001; Ziarek et al., 2011). The ectothermic life-history trait enables zooplankton to survive in a wide range of water temperatures and even in harsh environments, like deep-sea hydrothermal-vents (Hourdez et al., 2000). In addition, temperature can affect the physiology and behavior of zooplankton, for example, the swimming behavior of cladocerans (*Daphnia*) (Ziarek et al., 2011).

While many of the abiotic factors that influence zooplankton life-history are well established, much less is known about the biotic factors. Zooplankton is known to harbor many ecto- and endo-symbionts, including bacteria, protozoa, or viruses. It is hypothesized that these host-associated microbes may have symbiotic relationships (e.g., commensal, mutualistic, or parasitic characteristics) with their host (Qi et al., 2009). Despite its potential importance, the microbiome of freshwater zooplankton (e.g., copepods and cladocerans) is not widely explored. The importance of the microbiome was tested with a model organism (*Daphnia)* under laboratory conditions where the growth, survival, and reproduction of colonized daphnids performed significantly better than axenic ones (Sison-Mangus et al., 2015). It was also reported that specific bacterial taxa were consistently found in affiliation with *Daphnia*, even in geographically separated populations with different genetic backgrounds (Qi et al., 2009). Particularly, the genus *Limnohabitans* (β-proteobacteria) was a dominant constituent of the *Daphnia* microbiomes (Freese and Schink, 2011; Eckert and Pernthaler, 2014; Peerakietkhajorn et al., 2015; Callens et al., 2016). Other taxa also reported in microbiome studies with *Daphnia* were *Flavobacterium*, *Rhodobacter*, *Chromobacterium*, *Methylibium*, *Bordetella*, *Burkholderia*, and *Cupriavidus* (Qi et al., 2009). In freshwater copepods, it has been shown that both β-proteobacteria and Bacteroidetes were the most represented groups (Grossart et al., 2009).

The importance of epibionthic microbiomes in aquatic ecosystems and their link to biogeochemical cycling is still poorly understood. There are reports demonstrating that microbes attached to daphnids are more active than free-living ones (Eckert and Pernthaler, 2014). Moreover, there is a wealth of literature available showing higher activity and diversity by particle-associated microbes compared to free-living ones (LaMontagne and Holden, 2003; Mohit et al., 2014; Rieck et al., 2015). Nevertheless, it is still unknown whether microbiomes associated with zooplankton (i.e., copepods and cladocerans) differ from microbes associated with particles (PA) and free-living ones (i.e., bacterioplankton), and whether microbes exhibit similar or distinct lifestyles, metabolisms, or responses to environmental factors. To obtain more insight into these microbiomes in aquatic food webs and possible influencing factors, we used high-throughput 16S rRNA amplicon sequencing to assess diversity and composition of microbiomes associated with copepods, cladocerans, PA, as well as free-living bacterioplankton and how these are affected by warming and other environmental parameters. We investigated these by comparing the zooplankton associated and PA microbiomes as well as the free-living bacterioplankton in the water column of artificially warmed-lakes.

## Materials and Methods

### Description of Lakes

The Konin lakes mimic the thermal conditions of temperate lakes expected in the next 100 years (according to forecasts of IPCC – Intergovernmental Panel on Climate Change, 2013). The system of five lakes near the city of Konin in Poland (called here Konin lakes) are heated by two power plants, thereby offering a unique opportunity to study the effects of warming at whole ecosystem scale, mimicking future predictions of increased temperature. These lakes have been heated for over 60 years, which allows us to expect that populations inhabiting these ecosystems had enough time to acclimate, adapt or even evolve. The first power plant started to operate in 1958, the next one in 1967 (Socha and Hutorowicz, 2009; Tunowski, 2009). Both power plants take in water from the Konin lakes, use it in their cooling system, and discharge it back into the lake. All heated Konin Lakes are connected with channels creating two cooling circuits (Supplementary Figure S1). Warm water from the Konin power plant is discharged (channel depth: ∼2.3–2.5 m) (Ciemiński and Zdanowski, 2010) into lakes Licheńskie, Pątnowskie, and Mikorzyńskie. Heated water from the Pątnów power plant is released into Lake Gosławskie, and some of it is included in Konin power plant cooling phase (Tunowski, 2009). During summer (from May to September) water cools down in the large cycle, comprising lakes Licheńskie, Ślesińskie, Mikorzyńskie, Pątnowskie, and Gosławskie. The small winter cycle includes only lakes Licheńskie, Pątnowskie, Gosławskie, and southern part of Lake Mikorzyńskie. Due to heated water discharge, the temperature of Konin lakes is approx. 4–5°C higher in surface layer of deep stratified lakes (Ślesińskie, Mikorzyńskie, and part of Licheńskie) and in the whole water column of shallow lakes (Pątnowskie and Gosławskie) (Pyka et al., 2013; Stawecki et al., 2013).

### Sampling and Experimental Design

Zooplankton (copepods and cladocerans), PA, and bacterioplankton samples were collected from five heated lakes, Ślesińskie (SLE; 52°22′53.8″N 18°18′50.9″E), Mikorzyńskie (MIK; 52°19′58.6″N 18°18′32.5″E), Pątnowskie (PAT; 52°18′23.2″N, 18°16′20.2″E), Licheńskie (LICH; 52°18′24.1″N, 18°19′56.7″E), and Gosławskie (GOS; 52°17′27.1″N, 18°14′49.7″E), forming the cooling system of the power plants Konin and Pątnów (located in West Poland) at 25 (SLE, MIK, and PAT) and 27 (LICH, GOS) April, 2017 (Supplementary Figure S1). To avoid depth differences, 10 L water was sampled using a Uwitec water sampler (5 L) from the upper (1 m), middle (variable) and lower (variable) layers of each sampling station and mixed to have a composite sample (Supplementary Table S1). Zooplankton samples were collected from this composite sample using a 30-μm zooplankton net and stored in urine cups while the filtrates from the zooplankton net were stored in 50 mL falcon tubes for PA and bacterioplankton samples as well as chemical analysis. All samples were collected from 15 sampling stations across five lakes (selection of sampling stations were considered based on criteria like similar depth profile and avoidance of being directly in front of an inlet of cooling water to avoid mixing effects) and kept at 4°C until transfer to the lab. Using a stereo microscope, the animals were classified into four groups, Cyclopoid and Calanoid copepods, *Bosmina* and *Daphnia*, and sorted with sterile tweezers and washed twice with sterile WC media (Guillard and Lorenzen, 1972) known as standard medium for freshwater diatoms. The Cyclopoid and Calanoid copepods were sorted as copepods, and *Bosmina* and *Daphnia* were collected as cladocerans. Twenty live animals of each copepods and cladocerans groups were placed in a 2-mL tube. Distribution of copepods and cladocerans were described in Supplementary Table S2. Bacterioplankton samples were collected from 90-mL water subsamples onto 0.1-μm-pore-size filters (25-mm diameter) after pre-filtration through a 1.2-μm filter (GF/C, Whatman, United Kingdom). The 1.2-μm filters and the filtrates (90-mL) from 0.1-μm-pore-size filter were used for PA and chemical analysis, respectively. All animals, filters, and waters were stored at −80°C until further analysis.

### Water Physico-Chemistry

Depth, temperature, pH, electrical conductivity (EC), dissolved oxygen (DO) and oxidation-reduction potential (ORP), and Secchi disc visibility (SDV) were measured using multiparameter field probe (YSI 556 MPS) every 1-m depth from respective sampling station at the same time with the sampling (Supplementary Figure S2 and Supplementary Table S3).

### Nucleic Acid Extraction

Genomic DNA was extracted from 20 animals, 0.1-μm-pore-size filters and 1.2- μm filters using DNeasy PowerSoil Kit (Qiagen). Each 20 animals and small pieces of the filter, which was cut using sterile scissors, were placed into the PowerBead Tube of DNeasy PowerSoil Kit and further processed based on the manufacturer’s instructions.

### 16S rRNA Gene Amplicon Sequencing and Sequence Analysis

After DNA extraction of all samples, amplification of 16S rRNA gene was performed using universal bacterial primers 341F/799R targeting the V3 region of the 16S rRNA gene with a two-step barcoding approach according to a standard protocol (Herbold et al., 2015). Library preparation and sequencing were conducted by LGC Genomics GmbH^1^ according to the standard protocol, and libraries were paired-end sequenced (300 bp) using the Illumina MiSeq platform. Preliminary processing was carried out in Qiime (version 1.9.1) using default parameters (Caporaso et al., 2010). Sequences were clustered into Operational Taxonomic Units (OTUs) at 97% sequence similarity using the SILVA reference database (version 128) (Quast et al., 2012) and UCLUST (Edgar, 2010). For, assigning taxonomic classification BLAST analysis was done against the SILVA database (Altschul et al., 1990) in Qiime (version 1.9.1) using default parameters. Samples were then rarefied and randomly subsampled 10 times (using the Qiime command “multiple_rarefactions_even_depth.py”) to equal read-depths (3,000). All 10 OTU tables per sample were subsequently merged and exported for processing in R. In addition, UCLUST generated OTU-table was also compared with UPARSE method using “cluster_otus” command in USEARCH (version11) (Edgar, 2013) to compare both clustering approaches to estimate the alpha and beta diversity in all samples (Supplementary Figures S3, S4). All downstream analysis were performed in R (R Development Core Team, 2008) and described in detail in Supplementary Material.

### Statistical Analyses

All statistical analyses were performed in R version 3.6.0 (R Development Core Team, 2008) using the phyloseq (version 1.28.0) (McMurdie and Holmes, 2013), pvclust (version 2.0) (Suzuki and Shimodaira, 2006), vegan (version 2.5–5) (Oksanen et al., 2019), indicspecies (version 1.7.6) and edgeR (Robinson et al., 2010) packages. We used “envfit” function that fits environmental variables onto an ordination plot and provides an indication which environmental variables were significantly associated with ordination (Bray–Curtis distance measured from microbiome composition data). The significance of fitted environmental variables is determined using the permutation of environmental variables. In the plots, these results are displayed as correlations (*r*^2^) to microbiome compositions. Moreover, we have performed a “Mantel test” to determine the significant association between temperature and Bray–Curtis distances. To perform the above analyses, we considered the mean value of each environmental parameter (except lake depth where we considered the maximum lake depth) as metadata for microbial community analysis. Differential abundance analysis was performed in R using edgeR (Robinson et al., 2010) to elucidate which OTUs are significantly changed in abundance (log fold changes) from each microbiome in response to high and low temperatures. To perform this analysis, we have categorized all samples into two different groups, e.g., low temperature group (mean temperature of each lake <8°C) and high temperature group (mean temperature of each lake >8°C). Detailed descriptions for above analyses can be found in Supplementary Material.

## Results

### Bacterial Community Structure of Zooplankton, PA, and Bacterioplankton

**FIGURE 1:**
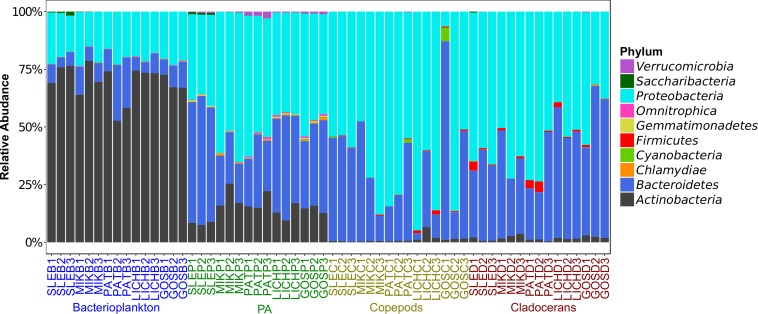
Relative abundance of top 10 phyla (>99% sequence reads) of microbial communities associated with zooplankton, particle-associated (PA), and free-living bacteria in five Polish heated lakes. Lakes’ names are shown as SLE, MIK, PAT, LICH, and GOS. The last letter and the digit of the sample names indicate microbiomes (B: bacterioplankton, P: PA, C: copepods, and D: cladocerans) and sampling stations of each lake (1, 2, and 3).

**FIGURE 2:**
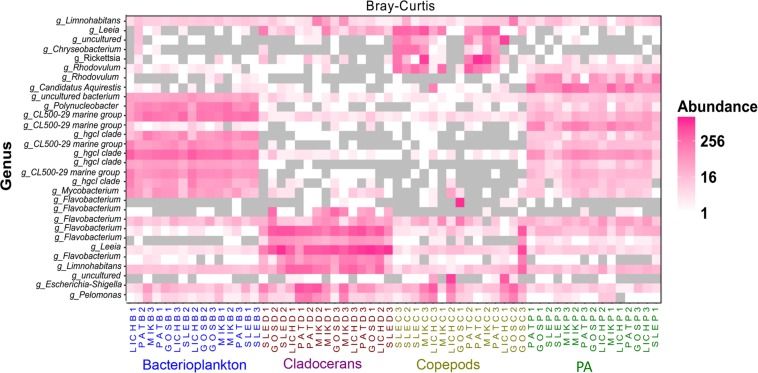
Heat map showing the top 30 most abundant OTUs (per 3000 sequence reads) across all microbiomes in five Polish heated lakes. Samples are ordered according to Bray–Curtis distance matrix within the same microbiome. Lakes’ names are shown as SLE, MIK, PAT, LICH, and GOS. The last letter and the digit of the sample names indicate microbiomes (B: bacterioplankton, P: PA, C: copepods, and D: cladocerans) and sampling stations of each lake (1, 2, and 3).

Proteobacteria and Bacteroidetes were the most dominant phyla in microbiome of zooplankton and PA (particle-associated fraction), representing ∼65 and ∼32% of total bacterial population at phylum level in copepods microbiome; ∼56 and ∼40% for cladocerans microbiome; and ∼48 and ∼34% for PA microbiome, respectively (Figure 1). In bacterioplankton, Actinobacteria (∼70% of total phyla) was the most dominant phylum followed by Proteobacteria (∼20%) and Bacteroidetes (∼10%). Relative abundance of Firmicutes was higher in zooplankton microbiome [cladocerans (∼1.3%) and copepods (∼0.5%)] as compared to bacterioplankton (∼0.02%) and PA (∼0.05%). At species or OTU level, relative species abundance differed among microbiomes (e.g., bacterioplankton, PA, copepods, and cladocerans) (Figure 2 and Supplementary Figure S5). The most abundant bacterial genera associated with zooplankton were *Flavobacterium*, *Leeia*, *Rickettsia*, *Escherichia*, *Limnohabitans*, and *Chryseobacterium*, whereas in bacterioplankton and PA, OTU’s of lineages without cultivated representatives *CL500-29 marine* group and hgcI-clade, as well as *Mycobacterium* and *Polynucleobacter* were most dominant. The difference in bacterial community structure among microbiomes (e.g., bacterioplankton, PA, copepods, and cladocerans) occurred in all sampled lakes (Adonis test, *F* = 20.7, *R*^2^ = 0.53, *p* < 0.001). A pvclust analysis (hierarchical clustering with *p*-values calculated via multiscale bootstrap resampling) confirmed two major clusters [100% AU (Approximately Unbiased) and 100% BP (Bootstrap Probability)] formed by different microbiomes of bacterioplankton and PA, and several small clusters formed by both copepods and cladocerans (Supplementary Figure S6).

### Bacterial Richness and Diversity of Zooplankton, Bacterioplankton, and PA

**FIGURE 3:**
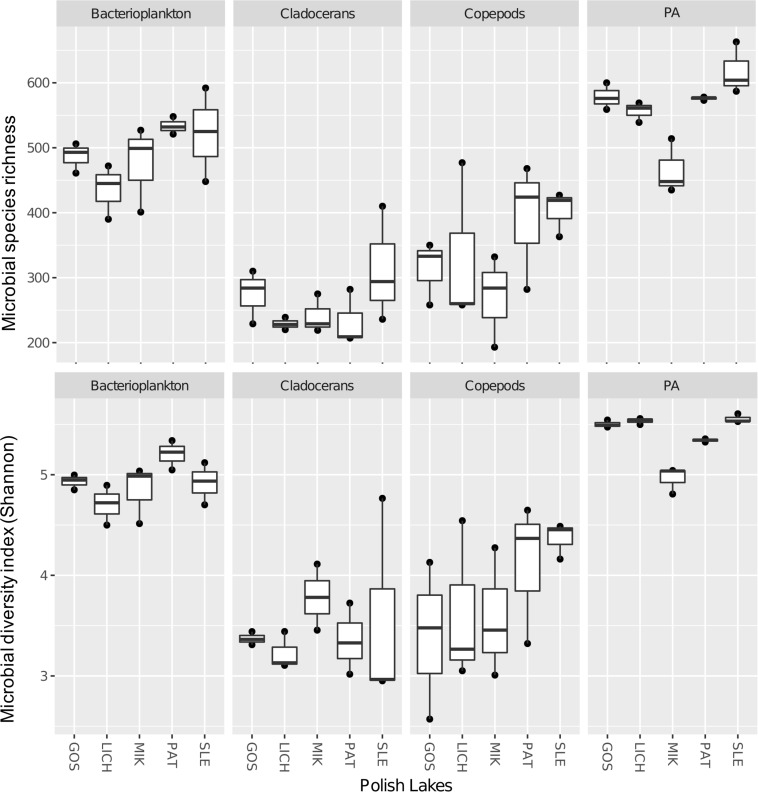
Microbial species richness (upper panel) and Shannon diversity index (lower panel) of microbial communities in five Polish heated lakes (SLE, MIK, PAT, LICH, and GOS).

Both richness and Shannon diversity were higher in bacterioplankton (1.4-fold for richness and 1.3-fold for Shannon diversity) and PA (1.6-fold for richness and 1.4-fold for Shannon diversity) as compared to copepods (Figure 3). Similar results were observed for cladocerans (i.e., bacterioplankton: 1.9-fold for richness and 1.4-fold for Shannon diversity; PA: 2.2-fold for richness and 1.5-fold for Shannon diversity). Paired *t*-test showed that both richness and Shannon diversity were significantly different across all biomes except between copepods and cladocerans, which had similar Shannon diversity index but different microbial richness (Supplementary Table S4).

### Indicator Species of Microbiomes

**FIGURE 4:**
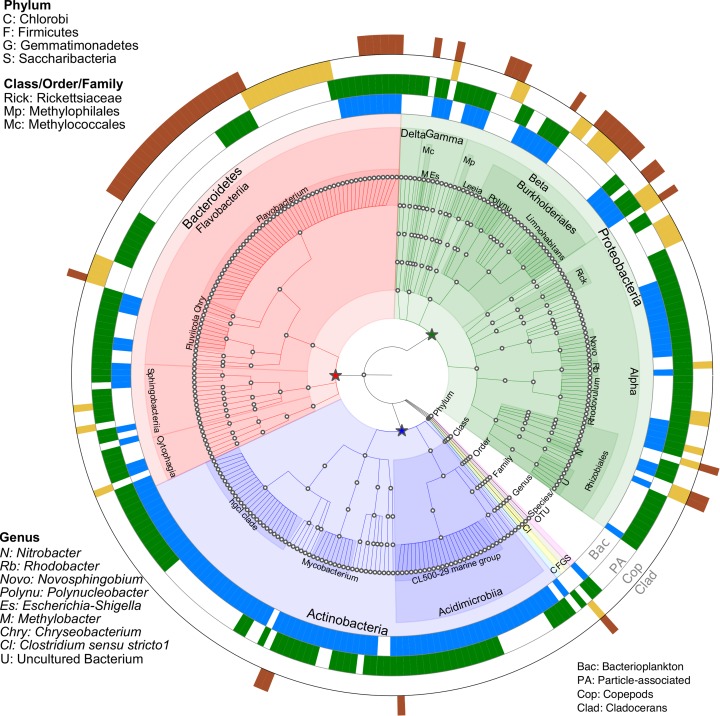
Taxonomic summary of OTUs responsive to microbiomes (Bacterioplankton, particle-associated (PA), Copepods, and Cladocerans) identified through Indicator species analysis (*p* < 0.05). The four outer rings represent the significant OTUs in response to microbiomes, with blank gaps indicating OTUs not significantly linked to microbiomes. Nodes on the tree (moving outward from center) correspond to taxonomic level (Domain, Phylum, Class, Order, Family, Genus, and Species/OTU). The star symbols (green, red and blue) indicate three major phyla (Proteobacteria, Bacteroidetes and Actinobacteria). Shaded areas of branches delineate defined taxonomic groups. See Supplementary Table S5 for full classification.

Indicator species analysis was performed to investigate which bacterial OTUs were significantly (*p* < 0.05) linked to a specific biome (Figure 4 and Supplementary Table S5). A total of 266 OTUs were identified as significant indicator species across all microbiomes. Compared to zooplankton, a larger number of OTUs (94) were linked to both bacterioplankton and PA, with 32 and 56 unique OTUs were linked to bacterioplankton and PA, respectively. With zooplankton, 23 unique OTUs linked to copepods and 22 unique OTUs linked to cladocerans while 11 OTUs were shared by both groups (Supplementary Table S5 and Supplementary Figure S7).

### Niche Differentiation of Biogeochemical Relevant Microbial Groups

**FIGURE 5:**
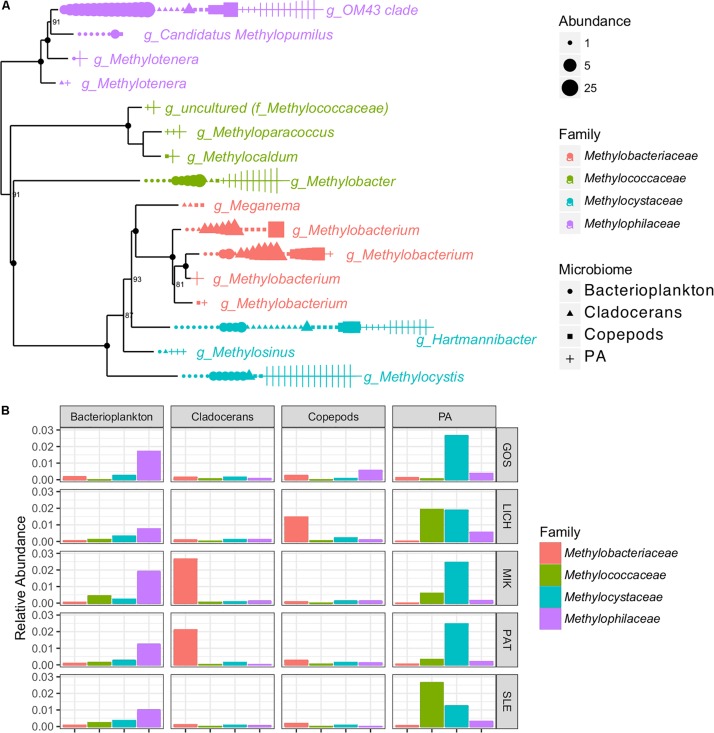
Phylogenetic tree based on 16S rRNA genes showing the OTU abundance (per 3000 reads) **(A)** and bar graph showing the relative abundance **(B)** of methane-oxidizing bacteria (MOB) and methylotrophs of four microbiomes in Polish lakes.

The indicator species analysis showed that at least two known OTUs were identified as belonging to the nitrogen (N) cycling genus, *Nitrobacter* which was found in bacterioplankton, copepods, and cladocerans. A large number of OTUs (16) representing methane-oxidizing bacteria (MOB) and methylotrophs were detected across all microbiomes (Figure 5). For example, several methylotrophic genera, including *Methylobacterium* and members of the OM43 clade were significantly associated with zooplankton while the methanotrophic genera *Methylobacter* and *Methylocystis* were significantly associated with PA (Figure 5).

### Relationships Between Environmental Parameters and Microbiome Compositions of Zooplankton, Bacterioplankton, and PA

**FIGURE 6:**
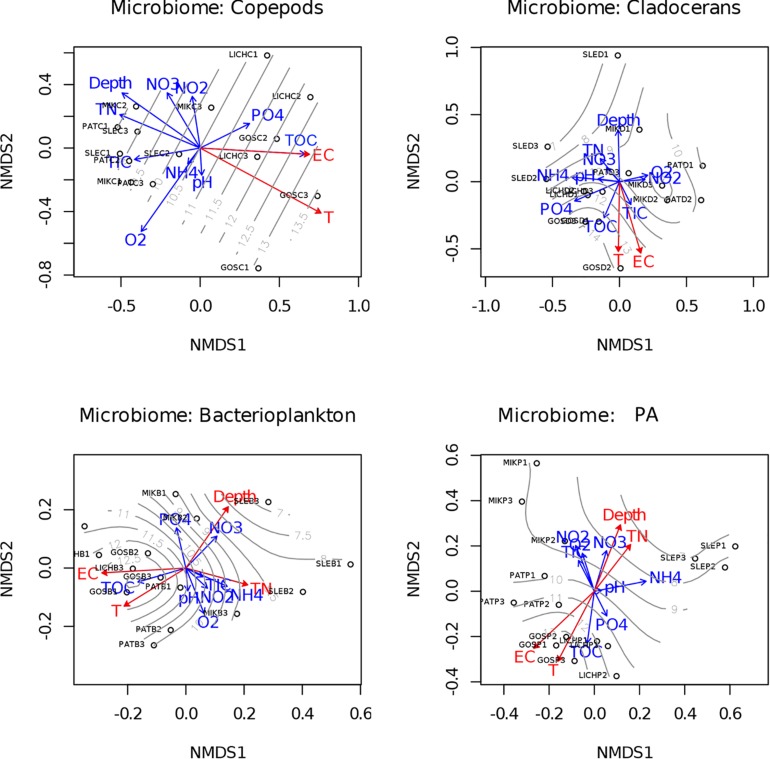
Relationship between bacterial community structure of microbiome and environmental parameters. NMDS ordination plots based on Bray–Curtis distance and fitted environmental parameters projected by “envfit” function showing relationships among samples based on OTU (97% sequence similarity) level changes in community composition. The significant (*p* < 0.05) environmental parameters are shown as red color. Gray-lines display the temperature gradient across lakes. T, temperature (°C); TN, total nitrogen; EC, electrical conductivity; TOC, total organic carbon; TIC, total inorganic carbon; O_2_; dissolved oxygen; NO_3_^–^, nitrate; NO_2_^–^, nitrite; NH_4+_, ammonium; PO_4_^2–^, Phosphate.

Our study lakes were interconnected and were artificially heated by power plants which generated a gradient of different temperatures across the studied lakes (see Supplementary Table S3 for all water chemistry data). To test which environmental parameters explained the variability observed in the microbiomes analyzed, the environment parameters (variables) were fitted onto an ordination plot using the “envfit” function (Non-metric multidimensional scaling (NMDS) with Bray–Curtis distance matrix generated from the OTUs) (Figure 6). The results showed that among the parameters measured, water temperature significantly correlated to the bacterial community composition of all microbiomes and of the free-living bacterioplankton (*r*^2^=0.61, *p* < 0.001 for copepods; *r*^2^ = 0.50 *p* < 0.05 for cladocerans; *r*^2^ = 0.6, *p* < 0.01 for bacterioplankton; *r*^2^ = 0.73, *p* < 0.01 for PA). In addition, the bacterial community composition of PA and bacterioplankton correlated to total nitrogen (TN), electrical conductivity (EC), and depth. Moreover, a Mantel test also supported our hypothesis that water temperature was significantly correlated to community structure of copepods (*r* = 0.35, *p* < 0.01), cladocerans (*r* = 0.24, *p* < 0.05), bacterioplankton (*r* = 0.24, *p* < 0.05), and PA (*r* = 0.44, *p* < 0.001).

### Differential Abundance of Species Between High and Low Temperature

**FIGURE 7:**
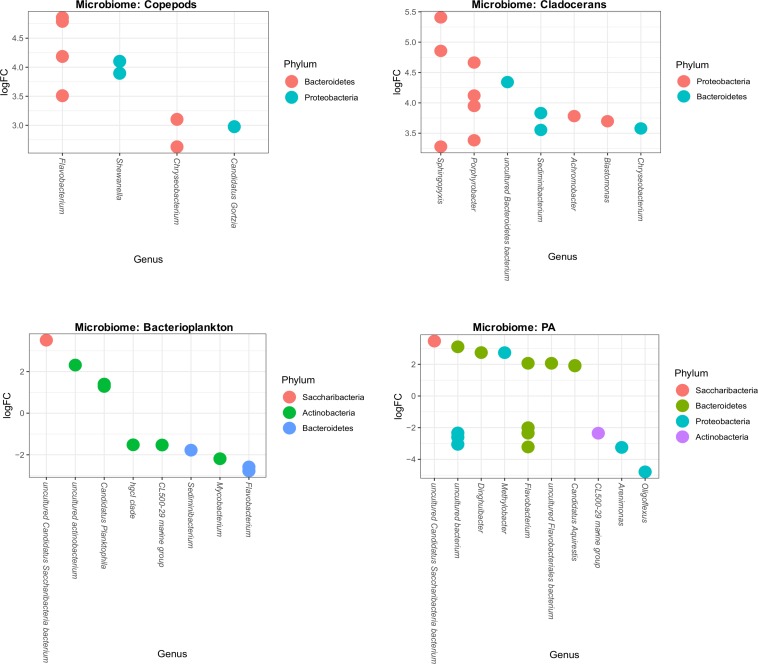
Differential abundance of OTUs of each microbiome (Copepods, Cladocerans, Bacterioplankton, and PA) in response to high and low temperatures. The *X*-axis shows the genus level classification and the *Y*-axis shows the log-fold changes (OTU abundance) due to temperature differences. Within each microbiome, the OTUs are visualized as colored-circles indicating different Phylum.

To assess which species differed in abundance between high and low temperature conditions, we used differential abundance analysis between each microbiome group of low and high temperature lakes. This analysis shows that some genera significantly increased or decreased their abundance (log-fold changes) across bacterioplankton and PA microbiomes. For example, 10 OTUs (8 genera) and 17 OTUs (10 genera) significantly increased or decreased in bacterioplankton and PA microbiomes, respectively (Figure 7). However, in zooplankton microbiomes, several genera significantly increased their abundance (log-fold changes). For example, in copepods microbiome, it shows 9 OTUs representing 4 genera (*Flavobacterium*, *Shewanella*, *Chryseobacterium*, and *Candidatus gortzia*) and in cladocerans microbiome, it shows 13 OTUs representing 7 genera (*Chryseobacterium*, *Sphingopyxis*, *Porphyrrobacter*, *Sediminibacterium*, *Achromobacter*, *Blastomonas*, and uncultured Bacteroidetes bacterium) significantly increased their abundance (log-fold changes) (Figure 7).

## Discussion

### Composition of Microbiomes of Zooplankton, PA, and Bacterioplankton

Microbes contribute significantly to the functioning of aquatic ecosystems, serving not only as food source for higher organisms but also act as catalysts for many biogeochemical reactions involved in nutrient-cycling, eutrophication, and greenhouse gas emissions. However, differences in microbial community structure, diversity, and functioning in various niches of the water column (e.g., microbiome of zooplankton and PA or bacterioplankton), especially under warmed conditions, are poorly investigated. Our results showed that host-associated (i.e., zooplankton) and free-living microbial communities are different, suggesting niche differentiation of microbes in various links of the aquatic food web. This differentiation was also obvious at higher taxonomic-level (e.g., phylum) where Proteobacteria and Bacteroidetes were dominant in zooplankton and PA, and Actinobacteria were dominant in bacterioplankton (i.e., free-living). A recent study showed a similar result in zooplankton-associated and bacterioplankton communities in marine environments (De Corte et al., 2018). Other microbiome studies of cladocerans (e.g., *Daphnia*) showed the presence of these two dominant phyla (Qi et al., 2009; Freese and Schink, 2011). In contrast, it has been shown that Firmicutes, Bacteroidetes and Actinobacteria are the most dominant members of the bacterial community associated with copepods in the North Atlantic Ocean (De Corte et al., 2014; Shoemaker and Moisander, 2017). This variation of the bacterial community associated with copepods may be caused by different trophic status (eutrophic lakes vs. oligotrophic marine environments) and seasonal differences (Shoemaker and Moisander, 2017). The phylum Bacteroidetes, especially the members of *Flavobacteria*, is highly abundant in the zooplankton-associated community which may play a crucial role in the degradation of high molecular weight organic matter (e.g., cellulose and chitin), proteins, and diatom debris (Qi et al., 2009; Buchan et al., 2014; Yang et al., 2015). The members of *Flavobacteria* are widely distributed in different host-associated samples, e.g., human gut (Bäckhed et al., 2005), fish gut (Banerjee and Ray, 2017; Egerton et al., 2018), and sponges (Parfenova et al., 2008; Hardoim and Costa, 2014). Another important role of *Flavobacteria* is that they can degrade cyanotoxins which eventually contribute to release of nutrients in freshwater ecosystems (Macke et al., 2017). Their presence in a large number of zooplankton groups probably suggest a symbiotic nature with a high diversification of metabolic traits (Cottrell and Kirchman, 2000; Beier and Bertilsson, 2013; De Corte et al., 2018), providing access to otherwise inaccessible nutrients (Macke et al., 2017).

### Microbial Alpha-Diversity of Zooplankton, PA, and Bacterioplankton

Microbial alpha-diversity (e.g., richness and Shannon index) was significantly lower in zooplankton (e.g., copepods, cladocerans) as compared to free-living bacterioplankton. This potentially indicates host-specialization of bacterial communities (Tang et al., 2010; De Corte et al., 2014, 2018) as the physico-chemical conditions of zooplankton differs from those in the ambient water (Tang et al., 2010). However, microbial abundance may differ on a per volume basis as a result abundance of zooplankton-associated bacteria can be higher than free-living bacteria (Tang et al., 2006, 2010). Apparently, being attached to particles is a suitable niche, taking the higher microbial richness and diversity of their associated microbiome into account. This may simply be caused by the fact that our size fractionation (1.2-μm) might not be entirely exclusive to phytoplankton but also can include other organisms and even larger particles can be included (i.e., protozoa, diatoms, sediments particles, and debris; and these containing their own microbial communities) contributing to higher microbial diversity.

### Indicator Species and Their Potential Link to Nutrient Cycling

The indicator species analysis identified numerous taxa that are present in one or more analyzed fractions. This finding gives rise to many open ecological research questions. For example, methanotrophic (utilizing methane) and methylotrophic bacteria (utilizing mainly methanol) were predominantly found in the PA and zooplankton fractions, respectively, indicating a disproportionate role of these fractions in C1 cycling in lakes. It is generally assumed that the bacterioplankton is the main driver for nutrient-cycling (e.g., C-cycling, N-cycling), however, recently zooplankton associated microbes have been shown to be more active in carbon cycling than their free-living counterparts (Eckert and Pernthaler, 2014) indicating modulation of pelagic biogeochemical activity by zooplankton associated bacteria. Methanotrophs and methylotrophs are groups of specialized microbes capable of using methane and methanol, respectively, (C1 cycling) as a sole carbon and energy source (Knief, 2015; Kallistova et al., 2017; Samad and Bertilsson, 2017). In this study, we observed that methanotrophic genera (e.g., *Methylobacter*, *Methylocystis*) predominantly occurred in the PA fraction. Recently, similar results were found in periphytic algae, explained by the authors as elevated methane abundance close to benthic methane sources in the littoral zone of Lake Mendota (Braus et al., 2017). In our lakes, pelagic phytoplankton associated methanotrophic presence maybe explained by methane production by algal associated methanogens as suggested by for Lake Stechlin (Grossart et al., 2011) which may attract and support methanotrophs. Although specific associations between methylotrophs and zooplankton have not been demonstrated till now, our observations may be explained by C1 compounds released by or produced in the guts of zooplankton (de Angelis and Lee, 1994) or by attraction to other compounds released by the zooplankton (e.g., nitrate, ammonium, or P) (Dennis et al., 2013). The bacterioplankton and the PA fractions, harbored another methylotrophic clade (e.g., OM43 clade). This OM43 clade (closely affiliated with the freshwater lineage of LD28) is widely distributed in both marine and freshwater environments and is responsible for the oxidation of C1 compounds (Newton et al., 2011; Jimenez-Infante et al., 2016). We also observed a large number of Actinobacterial clades (e.g., hgcI and CL500-29) that belonged to the microbiome of PA and bacterioplankton. The hgcI clade has ability to utilize carbohydrate as well as N-rich organic compounds from freshwater habitats (Warnecke et al., 2004; Liu et al., 2015). Surprisingly, CL500-29 clade is usually dominant in marine habitat but can also be observed in freshwater (Hugoni et al., 2017). There is no cultured representative of this CL500-29 clade, but enrichment experiments with seawater suggest that this group can utilize different carbon sources aerobically (McIlroy et al., 2017). *Limnohabitans* (β-proteobacteria) is responsible for dissolved organic carbon (DOC) flux (Eckert and Pernthaler, 2014) and these bacteria are widely observed in zooplankton indicating their potential symbiotic lifestyle although they also occur free-living (Peerakietkhajorn et al., 2015). Release of ammonium by zooplankton may also explain occurrence of N-cycling taxa, several taxa related to *alpha*-, *beta*-, and *gamma*-proteobacteria contribute to nitrification as well as denitrification (Peura et al., 2015). We identified two OTUs that belonged to the genera of *Nitrobacter* (class: alphaproteobacteria) which linked to N-cycling. *Nitrobacter* is responsible for the conversion of nitrite to nitrate which is an important pathway for nitrification (Mellbye et al., 2015; Daims et al., 2016; Jacob et al., 2017). A recent metagenomic study on zooplankton-associated bacterial communities in marine environment detected several genes (e.g., *nar*, *nor*, *nos*) which could be responsible for N-cycling, especially, nitrification, denitrification and dissimilatory nitrate, and nitrite reduction to ammonium (DNRA) (De Corte et al., 2018).

### Relationships Between Environmental Parameters and Microbiome Composition

We identified important environmental parameters that significantly correlated to microbiome composition of the lower-trophic levels of food webs (Figure 6). The bacterial community composition was significantly correlated to temperature and this was observed in host-associated microbiomes as well as free-living bacterioplankton. This supports our hypothesis that temperature has an important influence on shaping the microbiome structure of the lower-trophic levels of food webs. Changes in microbial community composition in response to warming was previously observed in bacterioplankton communities (Schindlbacher et al., 2011; Lara et al., 2013; Tang et al., 2015). However, no studies showed this response on host-associated microbes, especially, microbiome of zooplankton and PA. Additionally, our study also identified specific taxa that significantly increased or decreased in abundance (log fold changes) due to temperature differences (low vs. high). The increase or decrease of microbial abundance due to temperature varies across all microbiomes. Interestingly, the abundance of *Flavobacterium* (Phylum: Bacteroidetes) increased (>3-fold) in microbiome of copepods while declined (∼2-fold) in both bacterioplankton and PA microbiomes (except for two OTUs). Similarly, the abundance of *Chryseobacterium* (Phylum: Bacteroidetes) only increased (∼3-fold) in both copepods and cladocerans microbiomes. These results indicate that the increase of water temperature selects for only a few taxa likely based on their specific traits. At this stage, however, it is too early to speculate on the consequences of this shift in community composition for functioning of the hosts or of the ecosystem as a whole. Next to this, for the zooplankton related microbiome temperature induced changes in the host may also have led to selection for specific microbial taxa. A previous mesocosm study addressed this issue and observed that warming did not show any effects on zooplankton and other planktonic groups (Özen et al., 2012), yet, the microbiome of these groups was not assessed. Next to temperature, electrical conductivity also significantly correlated with the microbial community composition in the lower-trophic levels of food webs. This may be the result of enhanced ion concentrations (e.g., salts and inorganic materials) in these lakes, ultimately correlating to the microbial community composition. These lakes are located in an industrial and urbanized area, and the lakes used to be polluted by power plants (dust and ashes) that were sources of calcium and magnesium oxides, sulfur trioxides, iron, and aluminum oxides (Hillbricht-Ilkowska and Zdanowski, 1989). Presently, the lakes are surrounded by busy roads and towns where salt is used for defrosting pavements and roads. This could be a major NaCl source in these lakes. Apart from temperature and EC, lake depth and TN significantly correlated to microbial community composition of bacterioplankton and PA, but not with the microbiome of zooplankton. This may be caused by the trophic position of zooplankton, actively foraging on low C/N food of which a part will be released, alleviating the N-limitation of the zooplankton associated microbes (Wang et al., 2009; Abdel-Raouf et al., 2012; Delgadillo-Mirquez et al., 2016).

## Conclusion

We demonstrate that there are consistent differences in microbiomes composition of free-living bacterioplankton, zooplankton, and PA microbial communities. The microbes occupying these niches in the water column and their community structures are significantly correlated to temperature, a fact that may have implications for aquatic food webs in light of global warming. We also showed that niche differentiation between zooplankton and PA by biogeochemically relevant microbial groups may modulate lake biogeochemistry, an observation which has to be supported by extended experimentation and geochemical rate measurements.

## Data Availability Statement

The 16S rRNA amplicon sequences are publicly available in the NCBI, SRA database (BioProject accession: PRJNA481577). The OTU table, r scripts and input files for 16S rRNA are available on GitHub (https://github.com/Sainur/Samad_Microbiome_2018).

## Author Contributions

HL, SC, and PB designed the experiments. HL, SC, MM-F, and PB helped in the sampling and measured all physicochemical and ecological parameters. HL and MM-F helped in preparing samples for 16S amplicon sequencing. MS helped in analyzing the data and prepared the draft manuscript. All authors were involved in the manuscript writing and revision process.

## Conflict of Interest

The authors declare that the research was conducted in the absence of any commercial or financial relationships that could be construed as a potential conflict of interest.

## Footnotes

1 www.lgcgroup.com
