# Supplementary material for: Niche Differentiation of Host-Associated Pelagic Microbes and Their Potential Contribution to Biogeochemical Cycling in Artificially Warmed Lakes
Source: Front Microbiol. 2020 Apr 23;11:582. doi: 10.3389/fmicb.2020.00582 (PMC7190982; doi:10.3389/fmicb.2020.00582)
Supplement: Supplementary file 1 [file Data_Sheet_1.pdf]

Supporting information for

Niche Differentiation of Host-associated Pelagic Microbes and Their Potential Contribution to Biogeochemical Cycling in Artificially Warmed Lakes

Md Sainur Samad, Hyo Jung Lee, Slawek Cerbin, Marion Meima-Franke, Paul L. E. Bodelier

Table of Contents:

**Supplementary Methods**	Page 2-3
**Supplementary Figures (S1-S7)**	Page 4-10
**Supplementary Tables (S1,S2,S4)**	Page 11-12
**References**	Page 13

Supplemental methods

Analysis of microbial community composition

The rarified biom file was exported from Qiime and then processed in R using the Phyloseq package (McMurdie and Holmes, 2013). To account for the multiple rarifications (10 total) abundances were first normalized by dividing by 10 followed by rounding values to whole integers using the *transform_sample_counts()* command. Taxa (OTUs) with less than 1 count were deleted using the *prune_taxa()* command. Both Shannon and richness (alpha diversity) were calculated using the *estimate_richness()* command.

To determine samples forming statistically significant groups, a cluster analysis was performed using the pvclust package (method = Ward; distance matrix = Bray-Curtis; bootstrap value, n = 1000) (Suzuki and Shimodaira, 2006). Significant groups (representing 95% confidence) were marked with boxes (red).

Indicator Species Analysis

Indicator species analysis was performed in R using the "indicspecies" package with a function called "IndVal.g" (e.g. multipatt(Community data table, groups, func = "IndVal.g", duleg=F, control = how(nperm=1000))) (De Cáceres, 2013). The low abundant taxa were not considered in this analysis (the taxa that were not observed more than 3-times in at least 10% of the samples were removed from this analysis). See Supporting Information Table S5 for more details. Figure 4 was visualized with the open source package (GraPhlAn). Details methods can be found in https://bitbucket.org/biobakery/mbta_graphlan.

Phylogenetic tree

Phylogenetic tree (Figure 5A) was constructed with a set of sequences representative of the OTUs, by default using FastTree (Price et al., 2009) in Qiime1 (Caporaso et al., 2010). The tree file from Qiime1 outputs was sorted (kept only Methano and Methylotrophs) in R using Phyloseq package (McMurdie and Holmes, 2013) and then visualized with "plot_tree" function.

Envfit analysis

The "envfit" (function fits environmental vectors onto an ordination) (Figure 6) was performed in R using the "vegan" package (Oksanen et al., 2019) with default settings. To perform this analysis, microbiome community composition data was analyzed for Nonmetric Multidimensional Scaling (NMDS) with Bray-Curtis as distance matrix (default) using the function "metaMDS". Finally, the result from ordination (NMDS) and environmental variables (env) are combined for "envfit" analysis (formula: envfit(ordination, env, permutation = 999).

Mantel test

To determine the significant association between lake temperature and microbiome compositions, we used "Mantel test" in R using the "vegan" package (Oksanen et al., 2019) with default settings. To perform this analysis, microbiome community data (OTU table) and water temperature data were transformed into distance matrices ("Bray-Curtis" for microbial community and "Euclid" for temperature data) with the function "dist". Finally, person correlation was done between two distances (formula: mantel(dist.matrix1, dist.matrix.2, method = "pearson", permutations=999)).

Differential abundance analysis

To determine the significant OTUs of each microbiome in response to high and low temperatures, we used exact test in R using the "edgeR" package (Robinson et al., 2010). To perform this analysis all low variance OTUs (varianceThreshold = 1e-5) were removed from the biom file and then we performed a binary test using "exactTest" function. The result was adjusted by FDR (BH correction and alpha <0.05).

R script

A complete R-script (markdown format) for the above analysis can be found in this Github link:
https://github.com/Sainur/Samad_Microbiome_2018/blob/master/R_script_Polish_lakes.Rmd
.

Figures (S1-S7)

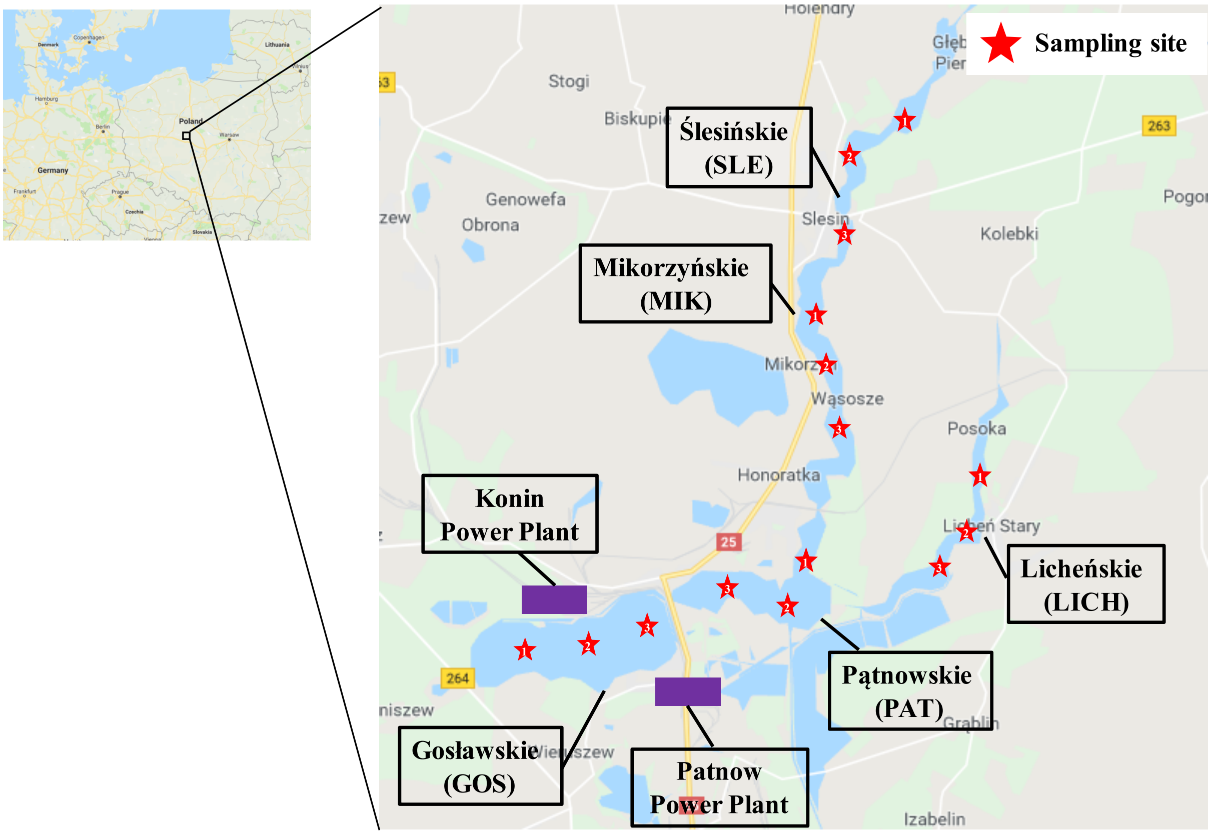

Figure S1. Location of five heated lakes in Poland, Ślesińskie (SLE; 52°22'53.8"N 18°18'50.9"E), Mikorzyńskie (MIK; 52°19'58.6"N 18°18'32.5"E), Pątnowskie (PAT; 52°18'23.2"N, 18°16'20.2"E), Licheńskie (LICH; 52°18'24.1"N, 18°19'56.7"E) and Gosławskie (GOS; 52°17'27.1"N, 18°14'49.7"E).

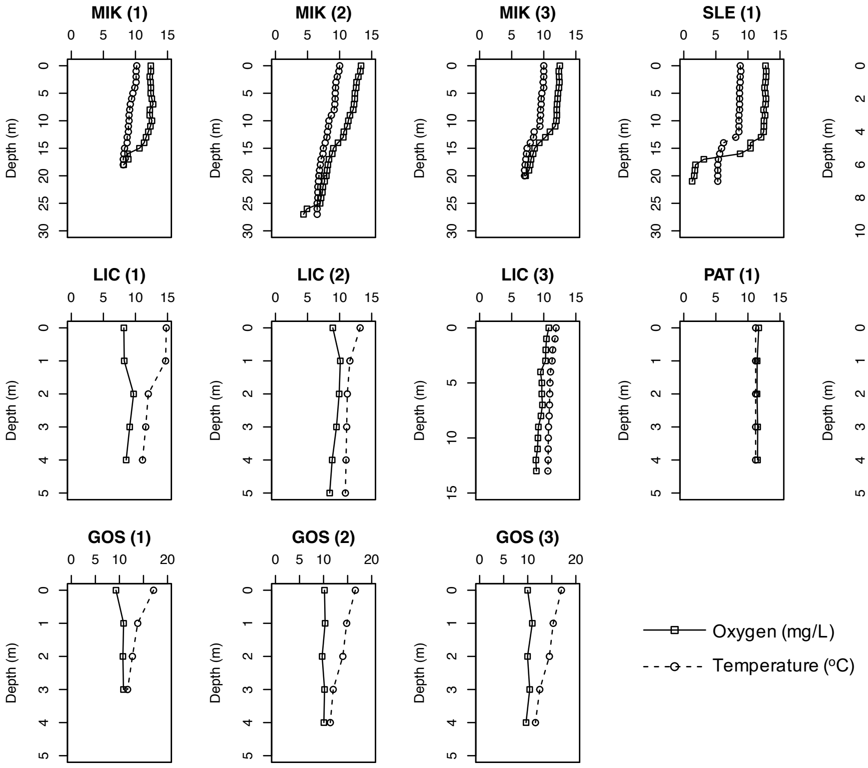

Figure S2. Temperature (°C) and dissolved oxygen (mg/L) profile of five Polish lakes. Lakes are indicated as SLE, MIK, PAT, LICH, and GOS.

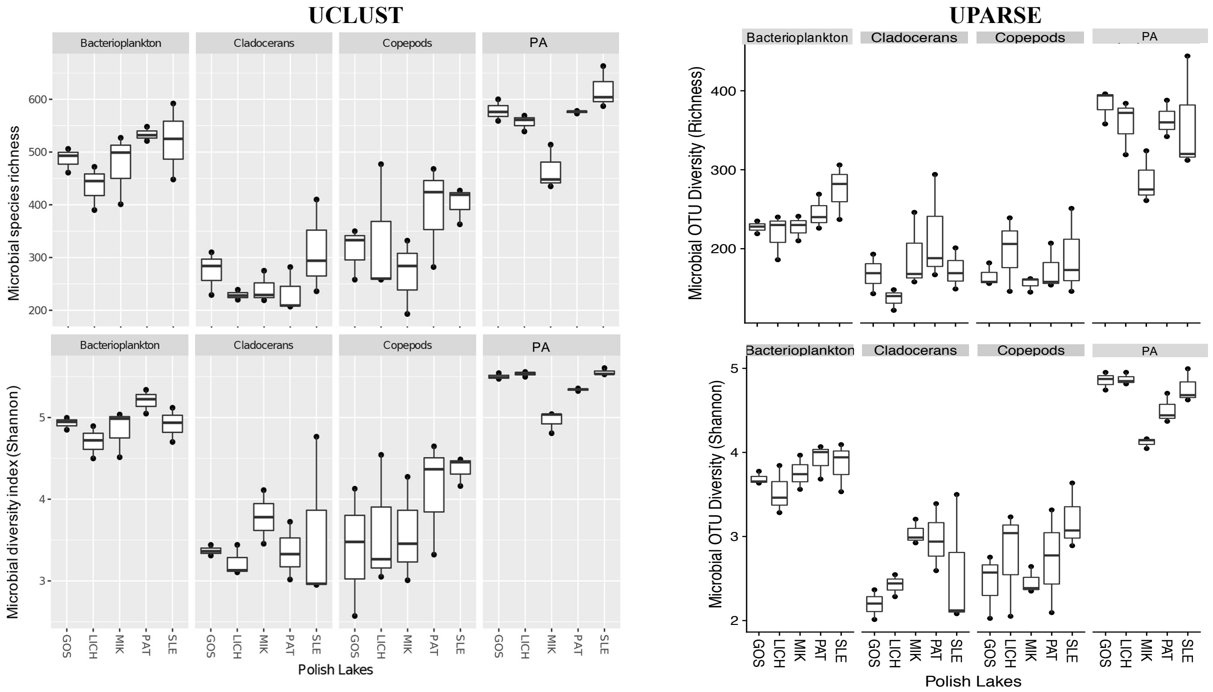

Figure S3. Comparison of microbial alpha-diversity measurements using two different OTU clustering approaches (UCLUST vs. UPARSE). Microbial species richness (upper left panel for UCLUST and upper right panel for UPARSE) and Shannon diversity index (lower left panel for UCLUST and lower right panel for UPARSE) of microbial communities in 5 Polish heated lakes (SLE, MIK, PAT, LICH, and GOS).

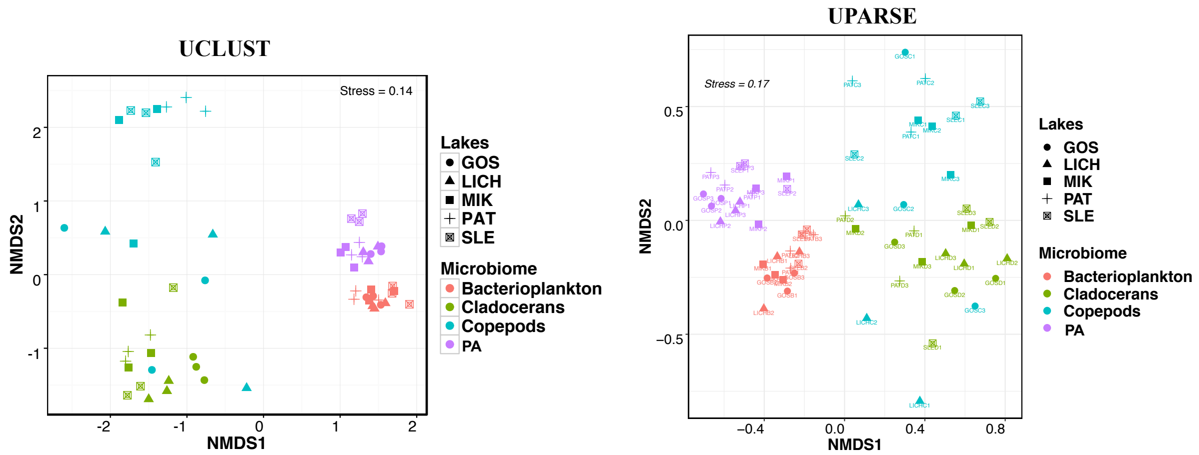

Figure S4. Comparison of microbial beta-diversity measurements using two different OTU clustering approaches (UCLUST vs. UPARSE). NMDS ordination plots are generated based on Bray-Curtis distance matrix.

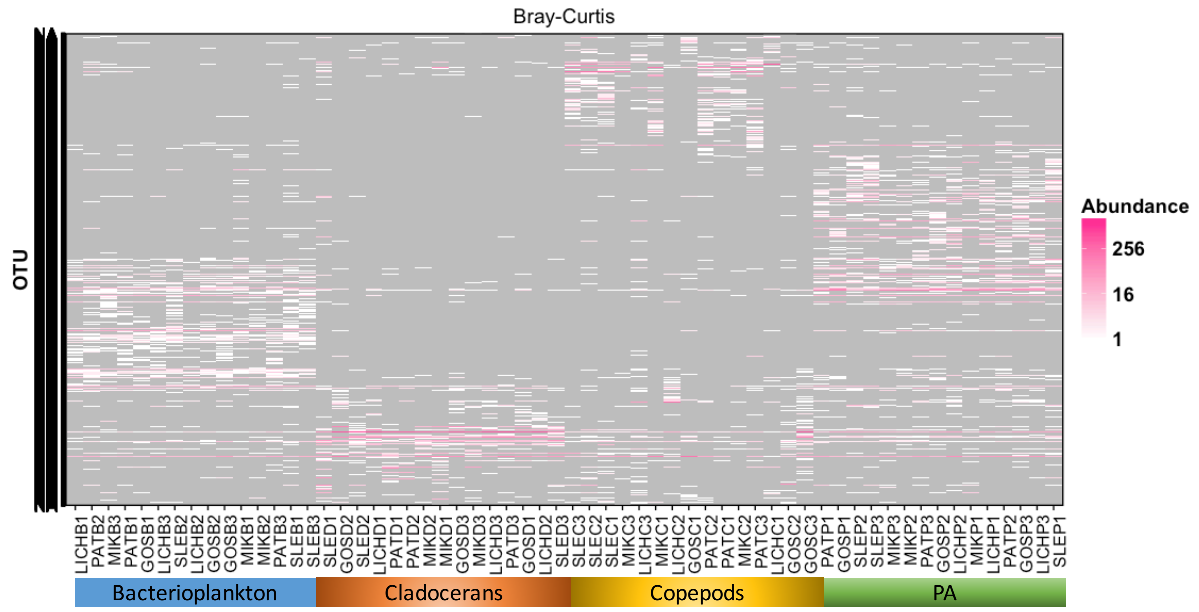

Figure S5. Heat map showing all OTUs across all microbiomes in 5 Polish heated lakes. Samples are ordered according to Bray-Curtis distance matrix within the same microbiome. Lakes are indicated as SLE, MIK, PAT, LICH, and GOS. The last letter and the digit of the sample names indicate microbiomes (B: bacterioplankton, P: particle-associated (PA), C: Copepods, and D: Cladocerans) and sampling stations of each lake (1, 2 and 3).

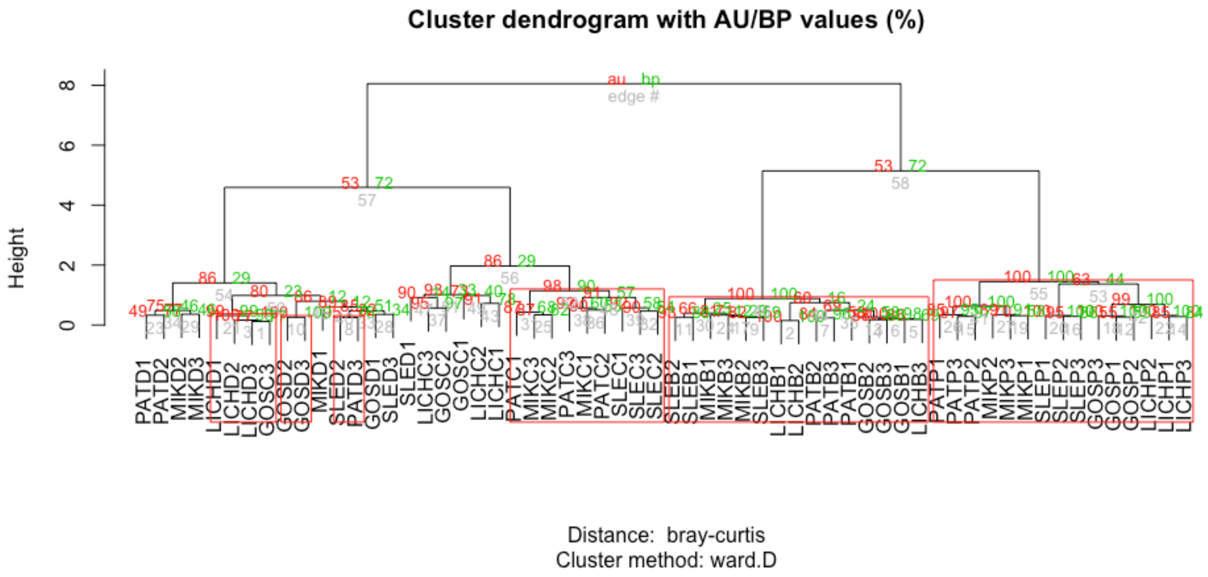

Figure S6. Pvclust tree displaying sample clustering based on Bray-Curtis distances calculated from 16S rRNA gene community composition and indicating significant clusters based on p values ([AU (approximately unbiased) BP (bootstrap probability)]) for each node. Red boxes mark clusters with 95% confidence. Bootstrap replication (n=1000). Lakes' names are shown as SLE, MIK, PAT, LICH, and GOS. The last letter and the digit of the sample names indicate microbiomes (B: bacterioplankton, P: particle-associated (PA), C: Copepods, and D: Cladocerans) and sampling stations of each lake (1, 2 and 3).

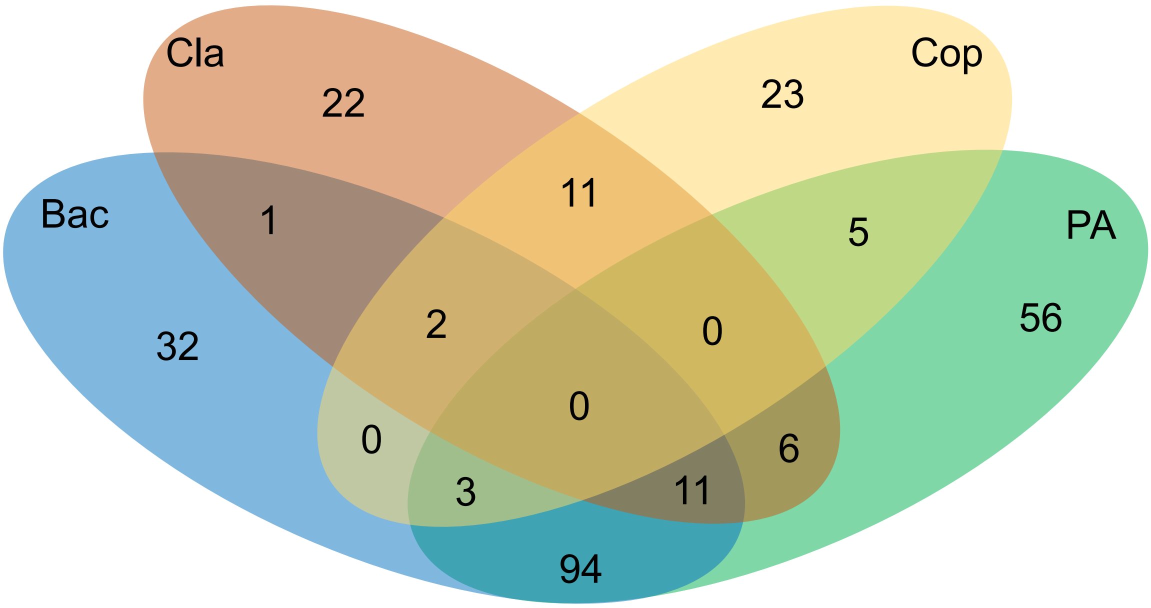

Figure S7. Venn diagram representing the number of OTUs that are associated with (unique and shared) microbiomes (Copepods [Cop], Cladocerans [Cla], particle-associated [PA], and bacterioplankton [Bac]).

Tables (S1, S2, S4)

Table S1. Depth information of the upper, middle and lower layers from five heated lakes. Sampling was done from the upper (1 meter), middle (variable) and lower (variable) layers of each sampling station.

Lake	Ślesińskie			Mikorzyńskie			Pątnowskie			Licheńskie			Gosławskie		
Station	(1)	(2)	(3)	(1)	(2)	(3)	(1)	(2)	(3)	(1)	(2)	(3)	(1)	(2)	(3)
Depth (m)															
Upper	1	1	1	1	1	1	1	1	1	1	1	1	1	1	1
Middle	9	5	12	8	14	10	2.5	2.5	2.5	2	2.5	6	2	2.5	2.5
Lower	18	10	24	17	28	21	3.5	3.5	4	3.5	3.5	12	3	4	4

Table S2. Distribution of cyclopoid and calanoid Copepods, *Bosmina* and *Daphnia* from five heated lakes.

		Ślesińskie	Mikorzyńskie	Pątnowskie	Licheńskie	Gosławskie
Copepods	Cyclopoid	20	15	20	15	20
	Calanoid	0	5	0	5	0
Clado-cerans	*Bosmina*	20	15	18	18	10
	Daphnia	0	5	2	2	10

Table S4. Paired t-test for mean comparison between microbiomes (Copepods, Cladocerans, PA and bacterioplankton). The significant ($p<0.05$) groups are shown as bold.

		Paired differences			t	df	p
			95% Confidence interval of the difference				
		Mean	Lower	Upper			
Shannon	Copepods-Cladocerans	0.35	-0.11	-0.11	1.64	14	0.123
	Bacterioplankton-Copepods	1.11	0.764	1.45	6.94	14	**0.000**
	Bacterioplankton-Cladocerans	1.46	1.19	1.72	11.97	14	**0.000**
	PA- Copepods	1.56	1.18	1.95	8.75	14	**0.000**
	PA - Cladocerans	1.92	1.58	2.25	12.43	14	**0.000**
	PA - Bacterioplankton	0.46	0.26	0.65	5.08	14	**0.000**
Richness	Copepods-Cladocerans	83.8	29.01	138.59	3.28	14	**0.005**
	Bacterioplankton-Copepods	148.8	103.02	194.58	6.97	14	**0.000**
	Bacterioplankton-Cladocerans	232.6	202.89	262.31	16.79	14	**0.000**
	PA - Copepods	217	173.03	260.97	10.58	14	**0.000**
	PA- Cladocerans	300.8	267.32	334.27	19.27	14	**0.000**
	PA - Bacterioplankton	68.2	35.44	100.96	4.46	14	**0.000**

References

Caporaso, J. G., Kuczynski, J., Stombaugh, J., Bittinger, K., Bushman, F. D., Costello, E. K., et al. (2010). QIIME allows analysis of high-throughput community sequencing data. *Nat Meth* 7, 335–336. doi:10.1038/nmeth0510-335.

De Cáceres, M. (2013). How to use the indicspecies package (ver. 1.7. 1). *R Proj* 29.

McMurdie, P. J., and Holmes, S. (2013). phyloseq: an R package for reproducible interactive analysis and graphics of microbiome census data. *PLoS ONE* 8, e61217. doi:10.1371/journal.pone.0061217.

Oksanen, J., Blanchet, F. G., Kindt, R., Legendre, P., Minchin, P. R., OHara, R. B., et al. (2019). vegan: Community Ecology Package. *R package version 2.5-5*. Available at: http://vegan.r-forge.r-project.org/.

Price, M. N., Dehal, P. S., and Arkin, A. P. (2009). FastTree: computing large minimum evolution trees with profiles instead of a distance matrix. *Mol. Biol. Evol.* 26, 1641–1650. doi:10.1093/molbev/msp077.

Robinson, M. D., McCarthy, D. J., and Smyth, G. K. (2010). edgeR: a Bioconductor package for differential expression analysis of digital gene expression data. *Bioinformatics* 26, 139–140. doi:10.1093/bioinformatics/btp616.

Suzuki, R., and Shimodaira, H. (2006). Pvclust: an R package for assessing the uncertainty in hierarchical clustering. *Bioinformatics* 22, 1540–1542. doi:10.1093/bioinformatics/btl117.
