# Supplementary material for: Niche Differentiation of Host-Associated Pelagic Microbes and Their Potential Contribution to Biogeochemical Cycling in Artificially Warmed Lakes
Source: Front Microbiol. 2020 Apr 23;11:582. doi: 10.3389/fmicb.2020.00582 (PMC7190982; doi:10.3389/fmicb.2020.00582)
Supplement: Supplementary file 2 [file Data_Sheet_2.pdf]

Table S3. Physico-chemical characteristics of the five Polish lakes (SLE, MIK, PAT, LICH, and GOS).

Sample ID	Replicates	Microbiome	Lakes	NO_3^- mg/L	NO_2^- mg/L	NH_4^+ mg/L	PO_4^{2-} mg/L	T °C	DO mg/L	pH	TN mg/L	TOC mg/L	TIC mg/L	EC µS/cm	Depth (max) m
GOSB1	1	Bacterioplankton	GOS	0	0	0.01	0.011	13.83	10.43	8.61	0.2747	2.781	25.13	642.95	3
GOSB2	2	Bacterioplankton	GOS	0	0	0	0.012	13.76	10.09	8.52	0.3931	2.208	44.02	647.96	4
GOSB3	3	Bacterioplankton	GOS	0	0	0.006	0.012	14.18	10.19	8.52	0.3572	2.085	38.04	644.08	4
LICHB1	1	Bacterioplankton	LICH	0	0	0	0.011	12.84	8.76	8.67	0.3618	1.485	28.48	641.04	4
LICHB2	2	Bacterioplankton	LICH	0	0	0.005	0.011	11.16	9.37	8.5	0.3049	5.307	16.44	638.52	5
LICHB3	3	Bacterioplankton	LICH	0	0	0.003	0.016	11.04	9.64	8.28	0.3357	6.436	25.97	653.29	13
MIKB1	1	Bacterioplankton	MIK	0	0	0.001	0.005	9.14	11.59	8.62	0.4327	1.167	38.76	616.98	18
MIKB2	2	Bacterioplankton	MIK	0.046	0.008	0	0.005	7.93	9.74	8.24	0.4914	1.316	39.22	634.93	27
MIKB3	3	Bacterioplankton	MIK	0.103	0.032	0	0.009	8.78	10.69	8.49	0.5052	2.176	32.84	620.76	20
PATB1	1	Bacterioplankton	PAT	0	0	0	0.008	11.2	11.51	8.59	0.4817	1.781	44.04	624.02	4
PATB2	2	Bacterioplankton	PAT	0	0	0	0.004	10.8	11.22	8.55	0.4024	1.839	34.97	626.1	4
PATB3	3	Bacterioplankton	PAT	0	0	0.008	0.004	10.16	11.1	8.55	0.5086	1.831	43.14	626.18	4
SLEB1	1	Bacterioplankton	SLE	0.003	0.002	0.007	0.002	7.53	9.81	8.48	0.4341	0.5684	18.92	578.91	21
SLEB2	2	Bacterioplankton	SLE	0.005	0	0.183	0.002	8.8	12.6	8.74	0.8777	2.408	44.47	574.02	9
SLEB3	3	Bacterioplankton	SLE	0.141	0.004	0.021	0.029	6.43	9.21	8.48	0.559	0.2806	44.92	595.49	26
GOSD1	1	Cladocerans	GOS	0	0	0.01	0.011	13.83	10.43	8.61	0.2747	2.781	25.13	642.95	3
GOSD2	2	Cladocerans	GOS	0	0	0	0.012	13.76	10.09	8.52	0.3931	2.208	44.02	647.96	4
GOSD3	3	Cladocerans	GOS	0	0	0.006	0.012	14.18	10.19	8.52	0.3572	2.085	38.04	644.08	4
LICHD1	1	Cladocerans	LICH	0	0	0	0.011	12.84	8.76	8.67	0.3618	1.485	28.48	641.04	4
LICHD2	2	Cladocerans	LICH	0	0	0.005	0.011	11.16	9.37	8.5	0.3049	5.307	16.44	638.52	5
LICHD3	3	Cladocerans	LICH	0	0	0.003	0.016	11.04	9.64	8.28	0.3357	6.436	25.97	653.29	13
MIKD1	1	Cladocerans	MIK	0	0	0.001	0.005	9.14	11.59	8.62	0.4327	1.167	38.76	616.98	18
MIKD2	2	Cladocerans	MIK	0.046	0.008	0	0.005	7.93	9.74	8.24	0.4914	1.316	39.22	634.93	27
MIKD3	3	Cladocerans	MIK	0.103	0.032	0	0.009	8.78	10.69	8.49	0.5052	2.176	32.84	620.76	20
PATD1	1	Cladocerans	PAT	0	0	0	0.008	11.2	11.51	8.59	0.4817	1.781	44.04	624.02	4
PATD2	2	Cladocerans	PAT	0	0	0	0.004	10.8	11.22	8.55	0.4024	1.839	34.97	626.1	4
PATD3	3	Cladocerans	PAT	0	0	0.008	0.004	10.16	11.1	8.55	0.5086	1.831	43.14	626.18	4
SLED1	1	Cladocerans	SLE	0.003	0.002	0.007	0.002	7.53	9.81	8.48	0.4341	0.5684	18.92	578.91	21

Sample ID	Replicates	Microbiome	Lakes	NO3	NO2	NH4	PO4	T	DO	pH	TN	TOC	TIC	EC	Depth (max)
SLED2	2	Cladocerans	SLE	0.005	0	0.183	0.002	8.8	12.6	8.74	0.8777	2.408	44.47	574.02	9
SLED3	3	Cladocerans	SLE	0.141	0.004	0.021	0.029	6.43	9.21	8.48	0.559	0.2806	44.92	595.49	26
GOSC1	1	Copepods	GOS	0	0	0.01	0.011	13.83	10.43	8.61	0.2747	2.781	25.13	642.95	3
GOSC2	2	Copepods	GOS	0	0	0	0.012	13.76	10.09	8.52	0.3931	2.208	44.02	647.96	4
GOSC3	3	Copepods	GOS	0	0	0.006	0.012	14.18	10.19	8.52	0.3572	2.085	38.04	644.08	4
LICHC1	1	Copepods	LICH	0	0	0	0.011	12.84	8.76	8.67	0.3618	1.485	28.48	641.04	4
LICHC2	2	Copepods	LICH	0	0	0.005	0.011	11.16	9.37	8.5	0.3049	5.307	16.44	638.52	5
LICHC3	3	Copepods	LICH	0	0	0.003	0.016	11.04	9.64	8.28	0.3357	6.436	25.97	653.29	13
MIKC1	1	Copepods	MIK	0	0	0.001	0.005	9.14	11.59	8.62	0.4327	1.167	38.76	616.98	18
MIKC2	2	Copepods	MIK	0.046	0.008	0	0.005	7.93	9.74	8.24	0.4914	1.316	39.22	634.93	27
MIKC3	3	Copepods	MIK	0.103	0.032	0	0.009	8.78	10.69	8.49	0.5052	2.176	32.84	620.76	20
PATC1	1	Copepods	PAT	0	0	0	0.008	11.2	11.51	8.59	0.4817	1.781	44.04	624.02	4
PATC2	2	Copepods	PAT	0	0	0	0.004	10.8	11.22	8.55	0.4024	1.839	34.97	626.1	4
PATC3	3	Copepods	PAT	0	0	0.008	0.004	10.16	11.1	8.55	0.5086	1.831	43.14	626.18	4
SLEC1	1	Copepods	SLE	0.003	0.002	0.007	0.002	7.53	9.81	8.48	0.4341	0.5684	18.92	578.91	21
SLEC2	2	Copepods	SLE	0.005	0	0.183	0.002	8.8	12.6	8.74	0.8777	2.408	44.47	574.02	9
SLEC3	3	Copepods	SLE	0.141	0.004	0.021	0.029	6.43	9.21	8.48	0.559	0.2806	44.92	595.49	26
GOSP1	1	PA	GOS	0	0	0.01	0.011	13.83	10.43	8.61	0.2747	2.781	25.13	642.95	3
GOSP2	2	PA	GOS	0	0	0	0.012	13.76	10.09	8.52	0.3931	2.208	44.02	647.96	4
GOSP3	3	PA	GOS	0	0	0.006	0.012	14.18	10.19	8.52	0.3572	2.085	38.04	644.08	4
LICHP1	1	PA	LICH	0	0	0	0.011	12.84	8.76	8.67	0.3618	1.485	28.48	641.04	4
LICHP2	2	PA	LICH	0	0	0.005	0.011	11.16	9.37	8.5	0.3049	5.307	16.44	638.52	5
LICHP3	3	PA	LICH	0	0	0.003	0.016	11.04	9.64	8.28	0.3357	6.436	25.97	653.29	13
MIKP1	1	PA	MIK	0	0	0.001	0.005	9.14	11.59	8.62	0.4327	1.167	38.76	616.98	18
MIKP2	2	PA	MIK	0.046	0.008	0	0.005	7.93	9.74	8.24	0.4914	1.316	39.22	634.93	27
MIKP3	3	PA	MIK	0.103	0.032	0	0.009	8.78	10.69	8.49	0.5052	2.176	32.84	620.76	20
PATP1	1	PA	PAT	0	0	0	0.008	11.2	11.51	8.59	0.4817	1.781	44.04	624.02	4
PATP2	2	PA	PAT	0	0	0	0.004	10.8	11.22	8.55	0.4024	1.839	34.97	626.1	4
PATP3	3	PA	PAT	0	0	0.008	0.004	10.16	11.1	8.55	0.5086	1.831	43.14	626.18	4
SLEP1	1	PA	SLE	0.003	0.002	0.007	0.002	7.53	9.81	8.48	0.4341	0.5684	18.92	578.91	21
SLEP2	2	PA	SLE	0.005	0	0.183	0.002	8.8	12.6	8.74	0.8777	2.408	44.47	574.02	9
SLEP3	3	PA	SLE	0.141	0.004	0.021	0.029	6.43	9.21	8.48	0.559	0.2806	44.92	595.49	26
